# The glycosyltransferase ST3GAL2 is regulated by miR-615-3p in the intestinal tract of *Campylobacter jejuni* infected mice

**DOI:** 10.1186/s13099-021-00437-1

**Published:** 2021-06-28

**Authors:** De Xi, Lukas Hofmann, Thomas Alter, Ralf Einspanier, Stefan Bereswill, Markus M. Heimesaat, Greta Gölz, Soroush Sharbati

**Affiliations:** 1 Institute of Veterinary Biochemistry, Freie Universität Berlin, Berlin, Germany; 2 Institute of Food Safety and Food Hygiene, Freie Universität Berlin, Berlin, Germany; 3 Charité - Universitätsmedizin Berlin, corporate member of Freie Universität Berlin, Humboldt-Universität zu Berlin, and Berlin Institute of Health, Institute of Microbiology, Infectious Diseases and Immunology, Berlin, Germany

**Keywords:** *Campylobacter jejuni*, Mucin, miRNA, Glycosyltransferase, Host cell response

## Abstract

**Background:**

*Campylobacter jejuni* (*C. jejuni*) infections are of increasing importance worldwide. As a typical mucosal pathogen, the interaction of *C. jejuni* with mucins is a prominent step in the colonisation of mucosal surfaces. Despite recent advances in understanding the interaction between bacterial pathogens and host mucins, the mechanisms of mucin glycosylation during intestinal *C. jejuni* infection remain largely unclear. This prompted us to identify relevant regulatory networks that are concerted by miRNAs and could play a role in the mucin modification and interaction.

**Results:**

We firstly used a human intestinal in vitro model, in which we observed altered transcription of MUC2 and TFF3 upon *C. jejuni* NCTC 11168 infection. Using a combined approach consisting of in silico analysis together with in vitro expression analysis, we identified the conserved miRNAs miR-125a-5p and miR-615-3p associated with MUC2 and TFF3. Further pathway analyses showed that both miRNAs appear to regulate glycosyltransferases, which are related to the KEGG pathway ‘Mucin type *O*-glycan biosynthesis’. To validate the proposed interactions, we applied an in vivo approach utilising a well-established secondary abiotic IL-10^−/−^ mouse model for infection with *C. jejuni* 81-176. In colonic tissue samples, we confirmed infection-dependent aberrant transcription of MUC2 and TFF3. Moreover, two predicted glycosyltransferases, the sialyltransferases ST3GAL1 and ST3GAL2, exhibited inversely correlated transcriptional levels compared to the expression of the identified miRNAs miR-125a-5p and miR-615-3p, respectively. In this study, we mainly focused on the interaction between miR-615-3p and ST3GAL2 and were able to demonstrate their molecular interaction using luciferase reporter assays and RNAi. Detection of ST3GAL2 in murine colonic tissue by immunofluorescence demonstrated reduced intensity after *C. jejuni* 81-176 infection and was thus consistent with the observations made above.

**Conclusions:**

We report here for the first time the regulation of glycosyltransferases by miRNAs during murine infection with *C. jejuni* 81-176. Our data suggest that mucin type *O*-glycan biosynthesis is concerted by the interplay of miRNAs and glycosyltransferases, which could determine the shape of intestinal glycosylated proteins during infection.

**Supplementary Information:**

The online version contains supplementary material available at 10.1186/s13099-021-00437-1.

## Background

The colonic mucosal layer is a dynamic and constantly renewing physical barrier providing protective functions through viscoelastic properties of mucins [1]. MUC2 is characterised by containing repeating domains rich in the amino acids proline, threonine and serine (PTS domains), which are densely *O*-glycosylated by the glycosylation machinery of the Golgi apparatus [2]. The PTS domain decorated with abundant *O*-glycans (mucin type *O*-glycans) forms a special “mucin domain” that makes up approximately 80% of the MUC2 mass and gives mucins extended conformation and furthermore, contributes to the structural integrity and complexity of mucins [2]. Assembled MUC2 polymers are stored in a condensed way in granules within goblet cells before being released into intestinal lumen. Once released, these large polymers are expanded in volume to form the mucus gel and provide the structural basis of the mucus layer [2]. Besides the mucins, also mucin-associated proteins such as the trefoil factor family (TFF) peptides are important for the restitution and integrity of the intestinal mucosal layer [3]. TFF3 is co-secreted with MUC2 by colonic goblet cells and usually acts in a cooperative manner with MUC2 ensuring elasticity and viscosity of MUC2 [3, 4].

The mucus layer provides protection from enteropathogens in several ways. This essential physical barrier hinders pathogenic as well as commensal bacteria to access the underlying intestinal epithelium. Previous studies have found that the lack of MUC2 and TFF3 resulted in pronounced defects in mouse intestinal homeostasis and enhanced susceptibility towards colitis, emphasising the importance of the mucus layer [1, 5, 6]. Moreover, the role of glycosylation of MUC2 in protecting the host from intruding bacteria has gained some appreciation in recent years. Application of knockout-based experiments led to reports of several glycosyltransferases involved in the biosynthesis and structural formation of core *O*-glycans of MUC2. For example, mice deficient in *N*-acetylglucosaminyltransferase (GCNT2) displayed an overall altered mucin composition and exhibited a defective mucus barrier function, given that GCNT2 initiates core 2 derived *O*-glycan branching which is the basis of mucin core structure 2 and 4 [7]. This indicates that *O*-glycosylation of MUC2 has important implications in forming the skeleton of the mucus layer and maintaining integrity of mucus barrier. Nevertheless, the mechanisms that regulate post-transcriptional modification of *O*-linked glycans on mucins like sialylation or fucosylation remain still unclear.

*O*-glycans are oligosaccharide moieties abundant on the cell surface proteins of both host and pathogens forming a critical interface with the biological milieu and profoundly influence the pathogen–host interaction [8, 9]. *O*-glycans on mucin proteins are used as ligands for bacterial adhesins and can prevent bacteria to access epithelial cells by binding and hampering them in mucus [10]. On the other hand, specific binding via “glycan-glycan talk” constitutes an important mechanism for bacteria to mediate adhesion and invasion of host cells [8]. During infection, bacterial pathogens can mediate host cell glycan modification utilising a broad range of glycosyltransferases and glycosidases, which in turn facilitates host adaption and adhesion and promotes access to glycans as carbon sources [8, 11]. *Campylobacter jejuni* (*C. jejuni*) is a major cause of bacterial food-borne disease worldwide and capable to induce gastroenteritis and irritable bowel disease in humans [12]. In the course of colonisation, the binding of *C. jejuni* to the host mucins is mediated by glycan-glycan interaction [8, 11]. Nevertheless, only little is known about the mechanisms of *C. jejuni* interaction with glycans of host mucosal surfaces and corresponding regulatory pathways involving mucin-modifying enzymes such as glycosyltransferases.

Recent efforts in non-coding RNA (ncRNA) research have expanded our understanding of mechanisms that regulate gene expression. MicroRNA (miRNA) belong to a well-studied class of ncRNAs, which can regulate gene expression through binding to complementary target sites of e.g. messenger RNAs (mRNAs), thereby initiating their degradation or suppression of translation [13]. Based on the fact that a single miRNA can target different genes and a single target gene can be coordinately regulated by different miRNAs, complex regulatory networks are formed to concert diverse biological processes including cell differentiation, proliferation, apoptosis or immune response [13, 14]. Our work as well as that of other research groups showed that miRNAs play a significant role in bacterial infections [14–17]. For example, the miR-125 family was shown to play an important role in immune response to bacterial as well as viral infections. Increased expression of miR-125b induced by lipomannan from virulent *Mycobacterium tuberculosis* can destabilise the transcript of tumour necrosis factor and therefore block its biosynthesis [17]. Moreover, work form Zhou et al. indicated that during *Helicobacter pylori* (*H. pylori*) infection, MUC2 expression could be post-transcriptionally affected by the cooperation of the lncRNA AF147447 with miR-34c [18]. Interestingly, Singh et al. found that under the influence of the endogenous microbiota, murine caecal miRNA signatures vary. Thus, intestinal barrier function may be affected by targeting genes that encode junctional proteins or glycosylation enzymes [19]. Furthermore, growing evidence has shown that miRNAs are major regulators of the glycome, controlling the level of glycan biosynthesis enzymes and playing a pivotal role in modulating and controlling glycosylation [20]. Kurcon et al. have utilised the glycogene target network of miR-200-family to identify three glycosylation enzymes (ST3 β-Galactoside α-2,3-Sialyltransferase 5 (ST3GAL5), ST6 *N*-acetyl-galactosaminide α-2,6-Sialyltransferase 5 (ST6GALNAC5) and β1,3-Glucosyltransferase (B3GLCT), controlling epithelial-to-mesenchymal transition in MDA-MB-231 cells [21]. Nevertheless, the number of validated glycogene-miRNA-interactions is still limited [20].

In this work, we applied in vitro and in vivo approaches to address which intestinal miRNA may be associated with MUC2 modification during *C. jejuni* infection and what potential regulatory networks exist in this context. Firstly, we confirmed that *C. jejuni* NCTC 11168 infection induced an altered transcription of MUC2 and the co-secreted peptide TFF3 in human intestinal epithelial cells in vitro. After in silico prediction of mucin-associated regulatory networks, we secondly examined the expression of two miRNAs and two glycosyltransferase genes in murine colonic tissue and found *C. jejuni* 81-176-mediated dysregulation of their transcription. Thirdly, we focused on the ST3 β-Galactoside α-2,3-Sialyltransferase 2 (ST3GAL2) and miR-615-3p interaction given their distinguished anti-correlated transcription upon *C. jejuni* 81-176 infection in vivo and were able to show that miR-615-3p interacts with ST3GAL2 targeting its cellular concentrations.

## Results

### *C. jejuni* infection alters the transcription of MUC2 and TFF3 in human intestinal epithelial cells in vitro

**Fig. 1:**
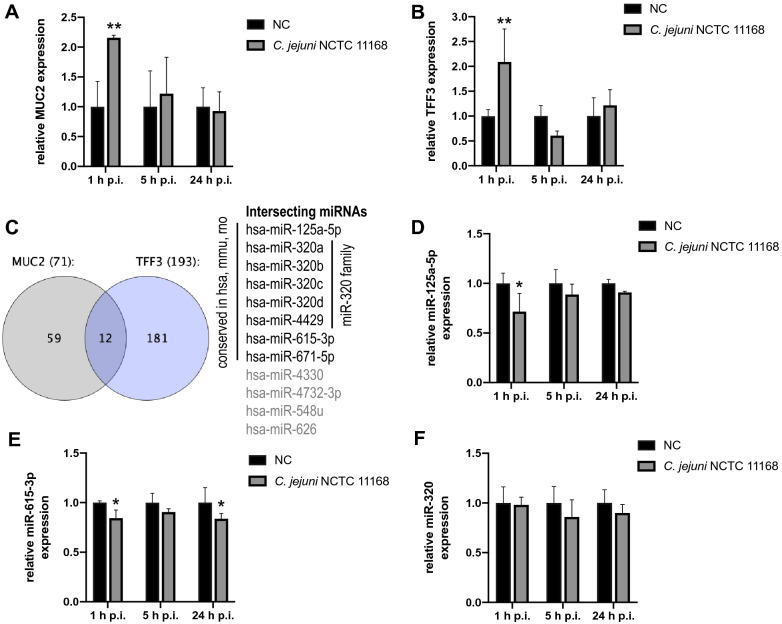
Prediction of MUC2-TFF3-associated miRNAs and relative mRNA expression of MUC2 and TFF3 along with relative expression of selected miRNAs in HT-29/B6 cells after *C. jejuni* NCTC 11168 infection.  **A**,** B** Fold changes of MUC2 and TFF3 mRNA expression were relatively calculated to non-infected controls and normalised with ACTB and B2M. **C** The Venn diagram shows the predicted numbers of MUC2-TFF3-associated miRNAs (12 miRNAs in the intersection). From those only miRNAs were selected that were conserved among human, mouse and rat. **D–F** Relative fold changes of miR-125a-5p, miR-615-3p and miR-320 at transcript level were calculated to non-infected controls and normalised with SNORD44 and SNORD47. Experiments were performed in triplicate and columns show the mean ± SD (bars). Asterisks represent a statistical significance compared to negative controls at each time point. *P ≤ 0.05, **P ≤ 0.01, unpaired *t*-test

MUC2 and TFF3 expression undergo changes during intestinal diseases caused by pathogenic bacteria, for instance [22]. In order to address this and find associated regulatory networks in response to *C. jejuni* infection, we determined the transcription of MUC2 and TFF3 in the human intestinal epithelial cell line HT-29/B6 following *C. jejuni* NCTC 11168 infection. The relative fold differences of MUC2 and TFF3 mRNA levels were calculated and revealed an approximately 2-fold up-regulation (*P* ≤ 0.01) of both factors relatively early (i.e., 1 h) post infection (p.i.) (Fig. 1A and B). Thereafter, a consistent decrease of MUC2 transcription was observed in the course of infection until 24 h p.i., whereas no significant differences could be assessed when comparing the infected and non-infected control groups at 5 and 24 h p.i. (Fig. 1A). Upon infection, the transcript level of TFF3 showed an early peak followed by a 0.61-fold decline at 5 h p.i. and a return to control levels until 24 h p.i. (Fig. 1B). Hence, both MUC2 and TFF3 mRNA expression significantly increased immediately after *C. jejuni* infection and subsequently decreased to levels that were comparable to non-infected controls.

### Prediction of MUC2-TFF3-associated miRNAs

Recent data [15, 16] showed that regulatory miRNA networks influence the host response to bacterial infections. Therefore, we speculated that besides the alterations in the transcription of MUC2 and TFF3 also post-translational modification of MUC2 might be changed which might be due to pathogen-mediated manipulation of regulatory networks composed of mRNA and miRNAs. To address this, we used miRmap [23] with a score of above 50—as described previously [24]—to identify miRNAs supposed to target MUC2 and TFF3, respectively. After intersecting both lists of miRNAs, we identified 12 miRNAs (Fig. 1C and Additional file 1) that could mutually target MUC2 and TFF3. From this, we selected eight miRNAs, which were conserved among human, mouse and rat. To assess their involvement in *C. jejuni* infection in vitro, we evaluated the expression of miR-125a-5p, miR-615-3p, miR-671-5p and miR-320 family in *C. jejuni* NCTC 11168 infected HT-29/B6 cells. The RT-qPCR results indicated that only miR-125a-5p and miR-615-3p showed regulated expression within 24 h p.i. Immediately after infection, miR-125a-5p was significantly downregulated (0.72-fold, *P* ≤ 0.05), whereas its expression returned to baseline values afterwards (Fig. 1D). MiR-615-3p expression showed a similar regulation with a 0.84-fold decrease (*P* ≤ 0.05) and its transcript level was rather consistent until 24 h p.i. (0.83-fold, *P* ≤ 0.05) (Fig. 1E). Both miRNAs possessed anti-correlated expression with MUC2 and TFF3 mRNAs. As shown in Fig. 1F, miR-320 expression was barely affected along the entire course of infection. Of note, the miR-671-5p level was below detection limit (data not shown).

### Identification of regulatory networks for in vivo analysis

**Fig. 2:**
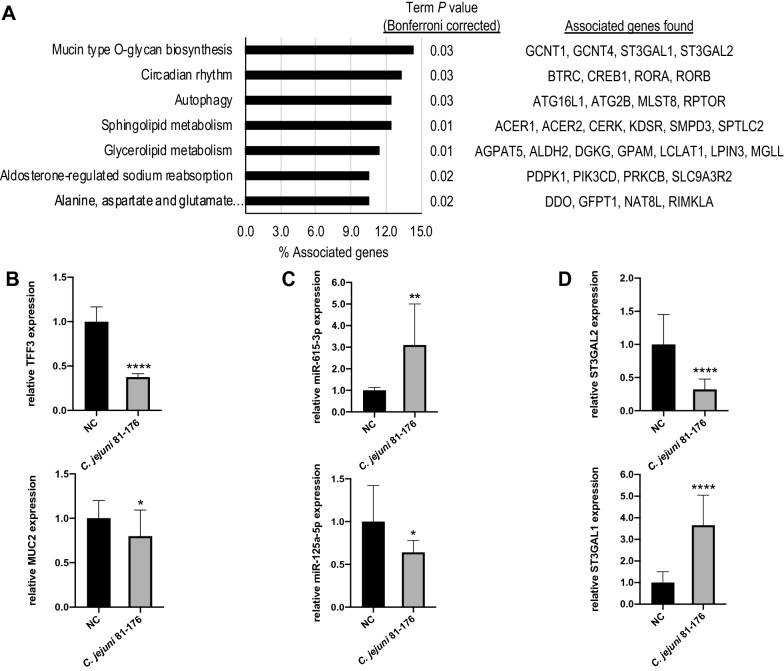
KEGG pathway enrichment with potential murine targets of miR-125a-5p and miR-615-3p and relative expression of mRNA and miRNA in colonic tissue of secondary abiotic IL-10^−/−^ mice upon *C. jejuni* 81–176 infection. **A** Gene enrichment analysis of mutual target genes of miR-125a-5p and miR-615-3p showing four significantly accumulated target genes (14.3% of associated genes) in the KEGG pathway ‘Mucin type *O*-glycan biosynthesis’ possessing a term *P* value corrected with Bonferroni step down of 0.03. **B** Fold changes of MUC2 and TFF3 mRNA expression were relatively calculated to naïve controls (NC) and normalised with HPRT and SDHA. **C** Expression levels of miRNA-615-3p and miR-125a-5p were calculated relatively to naïve controls (NC) and normalised with miR-16 and miR-145. **D** The relative transcript levels of ST3GAL1 and ST3GAL2 mRNA were calculated to naïve controls and normalised with HPRT and SDHA. The qPCR data were reported as the average of the relative expression in at least ten biological replicates; columns show the mean ± SD (bars). Asterisks represent a statistical significance compared to negative controls at each time point. *P ≤ 0.05; **P ≤ 0.01; ****P ≤ 0.0001, unpaired *t*-test

As shown above, an inverse correlation between two predicted miRNAs and MUC2 as well as TFF3 was observed in the early phase of *C. jejuni* NCTC 11168 infection in human cells. This indicated a regulatory importance of miR-125a-5p and miR-615-3p on related pathways upon *C. jejuni* infection. Therefore, we expanded our analysis to determine genes that are relevant to the post-transcriptional modification of MUC2 and might be regulated by the same miRNAs in the context of *C. jejuni* infection in vivo applying secondary abiotic mice [25]. For this purpose, we predicted the murine targets of both miR-125a-5p and miR-615-3p by means of another round of miRmap analysis (Additional file 1). After intersecting both lists of targets, we determined a gene list that was supposed to be regulated by both miRNAs. Using this list, we performed a KEGG pathway enrichment [26] by means of Cytoscape with the app ClueGO [27] (Fig. 2A, Additional file 2). In total, target genes were significantly assigned to seven KEGG terms and we found that four target genes were significantly (*P* ≤ 0.05) enriched in the KEGG pathway “mmu00512: namely, Mucin type *O*-glycan biosynthesis”, which corresponded to 14.3% of the associated genes in this term. They consisted of ST3 β-Galactoside α-2,3-Sialyltransferase 1 (ST3GAL1), ST3 β-Galactoside α-2,3-Sialyltransferase 2 (ST3GAL2), β1,6-*N*-acetylglucosaminyltransferase 1 (GCNT1), and β1,6-*N*-acetylglucosaminyltransferase 4 (GCNT4). These four glycosyltransferases were identified as potentially regulated candidates of both miRNAs upon *C. jejuni* infection in vivo (Fig. 2A).

### Expression analysis of interacting miRNAs and mRNAs in the colon of *C. jejuni* infected secondary abiotic IL-10^−/−^ mice

In addition to the early stage of infection with *C. jejuni* NCTC 11168 investigated in human cells, we further surveyed the later stage of infection with *C. jejuni* strain 81-176 in a well-established murine model [25] to assess the biological relevance of the observed regulations and predicted interactions in vivo. Therefore, the relative transcription of MUC2 and TFF3 was measured in colonic tissue samples taken at day 6 following peroral infection of secondary abiotic IL-10^−/−^ mice. As shown in Fig. 2B, C. *jejuni* 81–176 infection resulted in a pronounced decrease of TFF3 transcription when compared to naïve controls (0.38-fold change, *P* ≤ 0.0001), whereas the MUC2 transcription was slightly decreased upon infection (0.80-fold change, *P* ≤ 0.05). Secondly, we addressed whether the identified miRNAs were also regulated during *C. jejuni* 81-176 infection in mice, especially at the late stage of infection. As demonstrated in Fig. 2C, infected (secondary abiotic IL-10^−/−^) mice exhibited more than triple miR-615-3p transcript levels (*P* ≤ 0.01) compared to non-infected control mice. In contrast, the miR-125a-5p transcription decreased at the late stage of *C. jejuni* infection (0.64-fold changes, *P* ≤ 0.05; Fig. 2C). As pathway analysis predicted that glycosyltransferases involved in the mucin type *O*-glycan biosynthesis might be regulated by the identified miR-125a-5p and miR-615-3p, we were intrigued to unravel whether predicted targets ST3GAL1 and ST3GAL2 are dysregulated in an anti-correlated manner. Therefore, we examined their transcription in identical samples and observed a strongly reduced transcription (0.32-fold changes, *P* ≤ 0.0001) of ST3GAL2 elicited by *C. jejuni* 81–176 infection (Fig. 2D), which was anti-correlated with the miR-615-3p expression. In contrast, ST3GAL1 transcription was markedly enhanced (3.65-fold, *P* ≤ 0.0001) when compared to naïve controls (Fig. 2D) and exhibited inverse correlation with miR-125a-5p.

### ST3GAL2 is down-regulated in the murine epithelial cell line CMT 93 after RNAi using miR-615-3p mimic

**Fig. 3:**
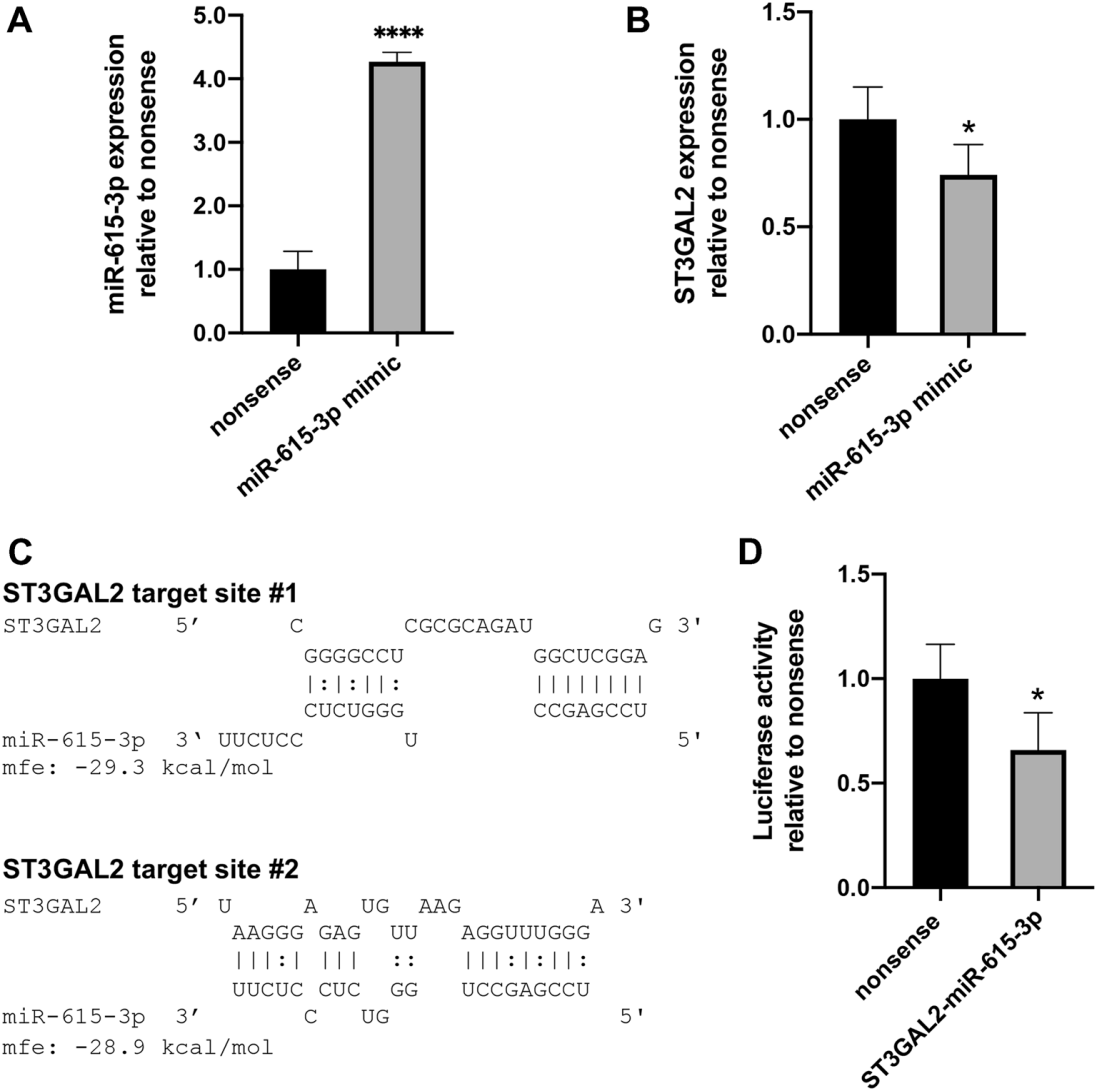
Specific interaction of miR-615-3p with ST3GAL2 confirmed by RNAi and dual luciferase reporter assay. **A**,** B** RT-qPCR analysis for ST3GAL2 transcription in CMT 93 cells transfected with miR-615-3p mimics and nonsense miRNA as control. Fold change of ST3GAL2 and miR-615-3p was calculated relatively to nonsense control and normalised with HPRT, SDHA, B2M and miR-16, miR-145, respectively. Datasets are presented as means of three biological samples and double measurements ± SD. **C** Identified target sites of ST3GAL2 and miR-615-3p were analysed with RNAhybrid. **D** Relative luciferase activity was determined to the nonsense miRNA mimic serving as control. Luciferase signal was assayed at 24 h post-transfection. Dataset was plotted as average values of at least four biological replicates with standard deviation from three measurements. Columns show the mean ± SD (bars). Asterisks represent a statistical significance compared to negative controls at each time point. *P ≤ 0.05, ****P ≤ 0.0001, unpaired *t*-test

MiRNAs are known to play a pivotal role in mRNA degradation or transcriptional repression by binding to their targets [13]. The results of the in vivo experiments suggested that the mRNA level of ST3GAL2 might be modulated by miR-615-3p and miR-125a-5p may play a regulatory role on ST3GAL1 transcription in colonic tissue after *C. jejuni* 81–176 infection. In this study, we only focused on the interaction of ST3GAL2 and miR-615-3p. To explore whether miR-615-3p was responsible for the decreased ST3GAL2 mRNA levels, we performed RNAi experiments using miR-615-3p mimics in the mouse rectum cell line CMT 93 considering nonsense transfected cells as controls. After RNAi, expression analysis of both miR-615-3p and its proposed target ST3GAL2 mRNA clearly revealed anti-correlated transcript levels, whereas the cellular miR-615-3p concentration was increased by 4.3-fold (*P* ≤ 0.0001) (Fig. 3A), which was accompanied by a significant 0.74-fold decrease in the ST3GAL2 mRNA level (*P* ≤ 0.05) (Fig. 3B).

### Reporter assays confirm ST3GAL2 as a target of miR-615-3p

To verify whether miR-615-3p directly binds to a target site within the 3′ UTR of ST3GAL2 and thereby repressively controls its post-transcriptional expression, we applied a dual luciferase reporter gene assay as described earlier [16]. Consequently, the 3′ UTR of murine ST3GAL2 was tested for binding sites of miR-615-3p using RNAhybrid [28] as previously described [3]. As shown in Fig. 3C, two interaction sites were identified. CMT 93 cells were transfected with a luciferase reporter plasmid containing a combination of both target sites [24] (Additional file 3), miR-615-3p mimic and a normalisation plasmid (pTKCluc). As shown in Fig. 3D, miR-615-3p mimics repressed luciferase activity by around 0.65-fold (*P* ≤ 0.05) compared to nonsense transfected controls. The 0.65-fold decrease in luciferase activity corresponded to the decrease in ST3GAL2 transcript levels after RNAi using miR-615-3p mimics in CMT 93 (approximately 0.75-fold of nonsense control). Hence, our results revealed specific interactions between at least one of the ST3GAL2 binding sites and miR-615-3p.

### ST3GAL2 protein levels in *C. jejuni* infected mice

**Fig. 4:**
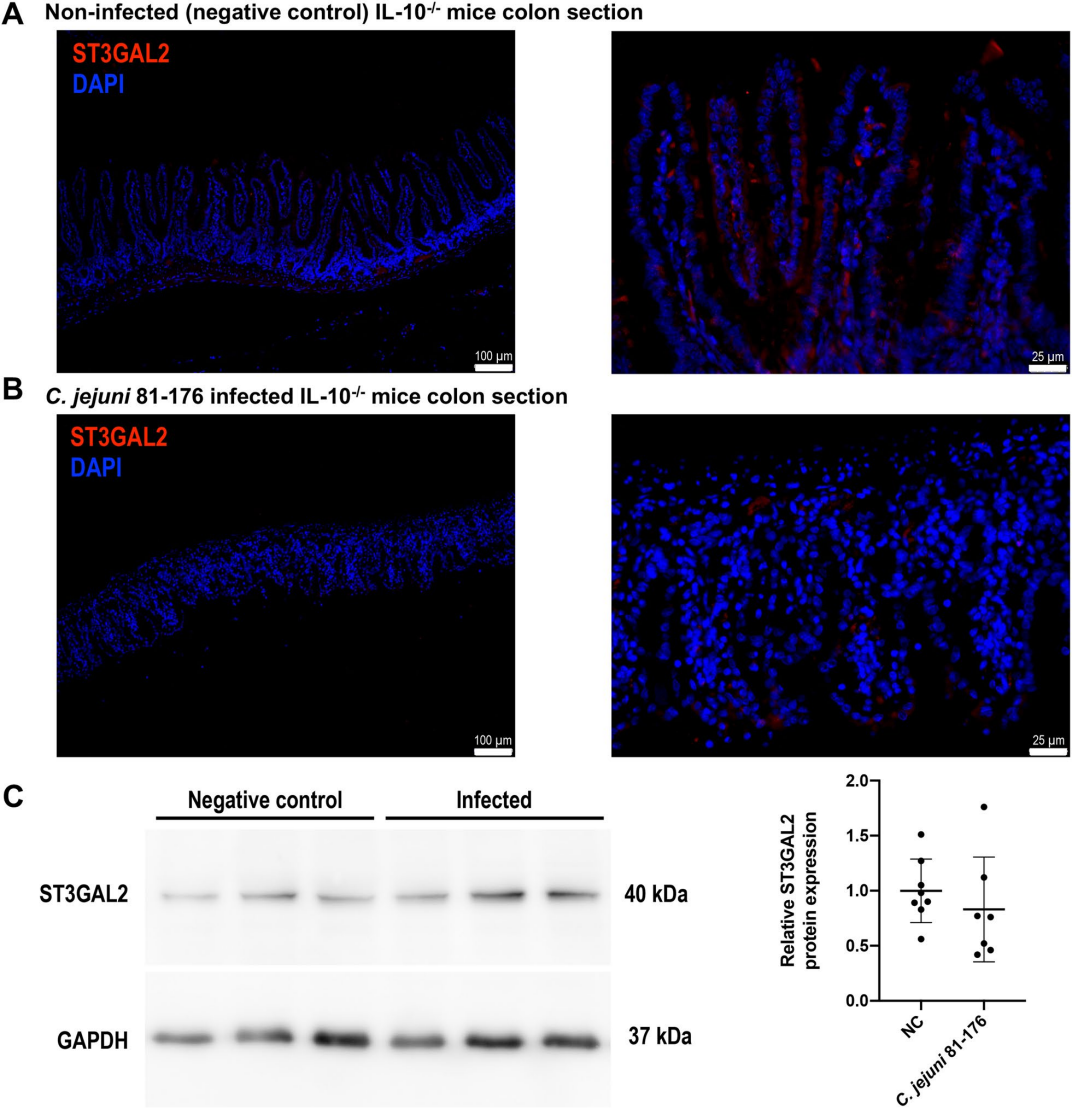
Detection of protein expression of ST3GAL2 in colonic tissue of secondary abiotic IL-10^−/−^ mice upon *C. jejuni* 81–176 infection. **A**,** B** Representative micrographs comparing ST3GAL2 immunofluorescence results in the colonic tissue of naïve mice (A) and *C. jejuni* 81-176-infected mice (B). ST3GAL2 was immunofluorescently labelled in red and nuclei was stained with DAPI in blue. Micrographs at left side are at magnification 10 × (Scale bar 100 μm) and right side at higher magnification 40× (scale bar 25 μm) and exposure time were the same in all sections. Data are representative for three biological replications. **C** Right panel exemplifies detection of ST3GAL2 from three individual replicates of infected and non-infected mouse colonic tissue via Western blotting. GAPDH is shown as respective loading reference. ST3GAL2 protein bands were quantified by densitometry relative to the respective GAPDH signals. Charts represent means of at least seven biological replicates ± SD, unpaired *t*-test

After showing that miR-615-3p specifically binds to the ST3GAL2 target sites in vitro and that intestinal ST3GAL2 transcript levels decrease upon *C. jejuni* infection in vivo, we further analysed the protein levels of ST3GAL2 by means of immunofluorescence and Western blot analyses of colonic tissue samples derived from *C. jejuni* infected secondary abiotic IL-10^−/−^ mice. As representatively shown in Fig. 4A and B and the Additional file 4, the immunofluorescent ST3GAL2 signal was much stronger in colon sections derived from non-infected as compared to *C. jejuni*-infected mice. However, the overall fluorescence signal in mouse colon sections stained with the anti-ST3GAL2 antibody was of low intensity. Furthermore, negative controls were analysed in serial sections on the same tissue area proving the specificity of detected signals (data shown in the Additional file 4). Moreover, *C. jejuni* infection of mice resulted in distinct histomorphological changes within the colon mucosa and lamina propria such as apoptosis of epithelial cells, villous blunting and irregular crypts as reported previously [29, 30].

In addition, we performed Western blot assays for ST3GAL2 using the same treated murine colonic tissue samples as for qPCR analysis. Compared to the significant decrease in ST3GAL2 expression at the mRNA level and the apparent decrease in immunofluorescence signal of the ST3GAL2 protein, we found only a slight trend towards down-regulation of ST3GAL2 at the protein level by Western blot in infected samples (not significant; Fig. 4C).

## Discussions

The intestinal mucus layer can confine enteropathogens in the outer layer and exclude pathogens through intestinal peristalsis and mucus turnover to protect epithelial cells from attack by pathogens [31]. Despite the defensive system the mucus provides, enteric pathogens are able to breach this barrier by employing manifold strategies. For instance, *C. jejuni* is able to grow and thrive in the mucus layer and can negotiate this barrier by distinct motility [32]. However, the host responses triggered because of *C. jejuni* interaction with the mucosa are not yet fully understood at the molecular level. Accordingly, the mechanisms of whether and how the mucin glycoprotein pattern is modulated during intestinal *C. jejuni* infection and what this means for the interaction with the host are still largely unknown.

The gel-forming mucin MUC2 and the viscosity regulating trefoil factor TFF3 are essential for intestinal integrity. It has been reported that microbial infection can induce mucin secretion by goblet cells as a host response to maintain and protect mucosal barrier [31]. Here, we observed significantly increased transcription of both MUC2 and TFF3 in cells derived from human colonic epithelial cell line HT-29/B6 immediately after *C. jejuni* NCTC 11168 infection. This is in agreement with a previous study, reporting increased transcriptional MUC2 expression upon *Escherichia coli* O157:H7 infection in human colon cells HT-29 [33]. In contrast to the upregulation of MUC2 and TFF3 mRNA in the initial stage of *C. jejuni* infection, we observed a slightly reduced transcription of MUC2 and profoundly decreased transcription of TFF3 in the colon of stably *C. jejuni* 81–176 infected mice (i.e., day 6 p.i.). Consistent with our observation, Bergstrom et al. found a slight reduction of MUC2 and TFF3 gene expression at 6 days p.i. and a significant decrease 10 days following murine *Citrobacter rodentium* infection [22]. According to our recent findings, *C. jejuni* strain NCTC 11168 and 81–176 exhibited comparable colonisation abilities and induced similar host responses with regard to histopathology and immune responses in secondary abiotic IL-10^−/−^ mice [34, 35]. Therefore, we assume that the observed dysregulation of MUC2 and TFF3 upon *in vitro* and *in vivo C. jejuni* infection was associated to the stage of infection rather than to the strains.

Evidence is emerging that miRNA-mediated regulation of mucins has important implications in inflammation and cancer biology [36]. For instance, miR-9 is responsible for gastric malignancies by targeting CDX2, conferring among others to enhanced proliferation of gastric cancer cells and MUC2 expression [36]. Further, miR-205 overexpression in CaCo-2 cells resulted in the accumulation of mucus-secreting goblet cell-like cells and enhanced mucin production and MUC2 expression [37]. Based on our analysis, anti-correlated expression of miR-125a-5p and miR-615-3p was associated with the dysregulation of MUC2 and TFF3 transcription at the early stage of *C. jejuni* NCTC 11168 infection *in vitro*. Likewise, we observed anti-correlated transcription of miR-615-3p with MUC2 and TFF3 mRNA upon established *C. jejuni* 81-176 infection *in vivo*. These findings suggest that the alterations of mucin expression induced by *C. jejuni* are connected with miR-125a-5p and miR-615-3p regardless of the strain. However, the direction of altered expressions seemed to be different at early and later stages of infection.

In this study, we aimed to build on the known role of MUC2 and TFF3 in intestinal bacterial infections and to identify novel regulatory aspects. For this purpose, we chose an approach that considers miRNAs as master regulators of those genes that belong to a metabolic or signalling pathway and interact accordingly. Here, we focused on the regulation of factors that are in the context of mucin modification and might be regulated by predicted miRNAs. Therefore, we concentrated our interest on target genes of miR-125a-5p and miR-615-3p, which may be related to metabolic changes of MUC2.

Upon enteric infection, quantity of host mucin may change by hypersecretion [31]. There can also be qualitative changes of mucins during infection including alterations in glycosylation. In this context, *H. pylori* was shown to change inflammation-associated mucin sialylation [31]. Previous studies have also observed altered degree of sulphation, sialylation and varied rate of glycosylation during intestinal inflammation as well as altered length and complexity of mucin glycans as a secondary effect to inflammation [38]. These alterations can further affect the adhesion of bacteria and the degradation of mucus by pathogens [38]. Thus, the interaction of mucus and pathogens seems to be a dynamic and complex process modulated by multiple factors [39]. Growing evidence has shown that miRNAs are major regulators of the glycome, playing a substantial role in modulating and controlling glycosylation [20]. However, our knowledge concerning the regulation of mucin modifying enzymes by miRNAs in bacterial infections is rather limited to date. According to our pathway analysis, several glycosyltransferases were potentially regulated by miR-125a-5p and miR-615-3p, from which we found two potential target genes (ST3GAL1, ST3GAL2) that are involved in mucin type *O-*glycan biosynthesis. *O*-glycans are the major glycans of mucins, which are generated via *O*-glycosylation and elongated or modified in a stepwise manner incorporating specific enzymes for sialylation, sulphation or acetylation [9]. ST3GAL1 and ST3GAL2 belong to a sialyltransferase family termed as ST3 β-Galactoside α-2,3-Sialyltransferase [40, 41]. ST3GAL1 predominantly adds sialic acid to the core 1 *O*-glycan [40], while ST3GAL2 transfers sialic acid to the terminal galactose residues found in glycoconjugates and it is also known for its importance in the biosynthesis of gangliosides [41]. Our RT-qPCR results showed increased ST3GAL1 mRNA expression in murine colonic tissue 6 days after *C. jejuni* infection, while a profound reduction of ST3GAL2 mRNA level was observed. This suggests that *C. jejuni* infection affects the expression of sialyltransferase genes in the mouse colon. This might occur in a miRNA-dependent manner, since predicted miRNAs (miR-125a-5p and miR-615-3p) possessed anti-correlated expression. However, whether observed miRNA-dependent alteration of sialyltransferases is only specific to *C. jejuni* infection requires more in-depth investigations. This will be the subject of our future research.

In the current study, we mainly focused on the interaction between ST3GAL2 and miR-615-3p, because on one hand, it showed typical binding properties by in silico analysis and on the other hand, our RT-qPCR analysis showed strongly negative correlation between both partners in colonic tissues derived from *C. jejuni* 81-176 infected mice. By both, the dual luciferase reporter assay and RNAi performed in CMT 93 cells, we demonstrated that miR-615-3p targets at least one predicted binding site within the 3′ UTR of the sialyltransferase ST3GAL2 specifically. Overall, the results suggest that miR-615-3p might play a regulatory role during *C. jejuni* infection of mice by targeting ST3GAL2. Nevertheless, the basic mechanism and regulatory pathway of miR-615-3p-ST3GAL2 interaction involved in sialylation upon murine *C. jejuni* infection still needs to be clarified. We also found that the transcription of ST3GAL1 and miR-125a-5p was negatively correlated. These observations could indicate a change in the mucin structure, as one sialyltransferase is down-regulated and the other up-regulated. However, whether miR-125a-5p specifically targets ST3GAL1 and how potentially altered glycosylation patterns impact the mucin structure are the questions remain to be investigated in our future studies.

In addition, we further determined the impact of *C. jejuni* 81-176 on ST3GAL2 expression at the protein level by performing immunofluorescent staining and Western blotting. Consistent with our results showing that infection caused down-regulation of ST3GAL2 mRNA, we observed reduced staining for ST3GAL2 proteins in murine colonic tissue samples at day 6 p.i. We also quantified changes of ST3GAL2 protein level via Western blotting and found a trend towards slightly decreased levels in the mouse colon 6 days after *C. jejuni* 81-176 infection. Nevertheless, the majority of our results reveal that the expression of the sialyltransferase ST3GAL2 is regulated during infection with *C. jejuni* 81-176 by miR-615-3p. Due to the fixation, we could not follow the mucin change by staining, as the mucus was dehydrated by paraformaldehyde fixation and thus differences e.g. in thickness could not be traced [42].

## Conclusions

In conclusion, using in vivo and in vitro approaches, this study demonstrated that *C. jejuni* infection induced dysregulation of the mucin MUC2 and its mediator TFF3 mRNA as well as mucin-associated miRNAs (miR-125a-5p and miR-615-3p) in an apparently strain-independent manner. Moreover, we observed aberrant transcription of sialyltransferases ST3GAL1 and ST3GAL2 that are involved in mucin type *O*-glycan biosynthesis. Our results suggest that these mucin-associated factors interact in a coordinated manner concerted by a miRNA-dependent regulatory network during *C. jejuni* infection. Herein, ST3GAL2 has been identified as a target of miR-615-3p *in vitro* and for the first time we show a regulatory relationship of miR-615-3p and ST3GAL2 involved in the host cellular response to *C. jejuni* 81-176 infection in mice. Whether the mechanisms underlying these alterations during *C. jejuni* infection involve processes that are dependent or coordinated with the regulation of other predicted miRNAs remain to be addressed. For further explorations, it could be intriguing to know the possible impact of miRNA-regulated sialyltransferases on mucin structure during *C. jejuni* infection. Moreover, we will shed light on the functional effects of miR-615-3p-ST3GAL2-interaction in response to other bacterial infections in future.

## Methods

### Bacterial strains, cell lines and culture conditions

*Campylobacter jejuni* NCTC 11168 were grown as described earlier [43]. Briefly, bacteria were routinely grown on Mueller-Hinton agar (Oxoid, Munich, Germany) supplemented with 5% defibrinated sheep blood or in Brucella broth (BD, Heidelberg, Germany) at 37 °C under microaerobic conditions (10% CO_2_, 6% O_2_ and 85% N_2_) generated by Anoxomat (Omni Life Science, Bremen, Germany). *C. jejuni* NCTC 11168 were grown to mid-exponential phase in BB and centrifuged (14,000×*g*, 5 min), and re-suspended in the cell culture medium prior to infection assay in vitro. For in vivo infection, a stock solution of *C. jejuni* 81–176 strain (stored in − 80 °C) was thawed and aliquots streaked onto Karmali agar (Oxid, Wesel, Germany) and incubated at 37 °C under microaerobic conditions for 48 h, as previously described [44]. Bacteria were harvested with a final inoculum of 10^9^ bacterial cells in sterile phosphate buffered saline (PBS) (Gibco, Life Technologies, UK) immediately prior to peroral infection of mice.

The subclone HT-29/B6 [45] of the human colorectal adenocarcinoma cell line HT-29 (DSMZ_ACC299) was maintained as previously described [43]. Briefly, HT-29/B6 cells were routinely cultured in RPMI 1640 medium (Lonza, Basel, Switzerland) supplemented with 10% (v/v) FCS superior (Biochrom, Berlin, Germany) in 75 cm^2^ tissue culture flasks (Sarstedt, Nümbrecht, Germany) at 37 °C and 5% CO_2_ under a humidified atmosphere until a confluence of approx. 80 % was reached. For infection assays, 5 × 10^5^ HT-29/B6 cells were seeded into each well of a 6 well plate (Sarstedt) and incubated for 7 days with changing the media regularly. Murine rectum carcinoma cell line CMT 93 (ECACC 89111413) was cultured as described by Jonckheere et al. [46]. Briefly, CMT 93 was maintained in high glucose (4.5 g/L) Dulbecco’s modified Eagle medium (DMEM, Lonza, Köln, Germany) supplemented with 10% (v/v) fetal bovine serum (FBS, Biochrom, Cambridge, United Kingdom). Cultivation of cells was performed in 75 cm^2^ tissue culture flasks (Sarstedt, Nümbrecht, Germany) at 37 °C in a humidified 5% CO_2_ atmosphere until a confluence of approx. 75% was reached. For nucleofection, 1 × 10^6^ CMT 93 cells were taken from pre-cultured CMT 93 cells with 75% confluency, followed with transfection and 24 h incubation.

### Animal experiments and tissue sampling

Mouse experiments were carried out in compliance with the European Guidelines for animal welfare (2010/63/EU). The protocol was approved by the commission for animal experiments headed by the “Landesasmt für Gesundheit und Soziales” (LaGeSo, Berlin, registration numbers G0172/16 and G0247/16). Animal welfare was monitored twice daily by assessment of clinical conditions. As previously described [30], secondary abiotic IL-10^−/−^ mice (C57BL/6j background) from the Forschungseinrichtungen für Experimentelle Medizin (FEM, Charité-University Medicine Berlin) were included in the study. Infected animals (in total n = 12) were perorally challenged with 0.3 ml PBS containing 10^9^ colony forming units (CFU) of *C. jejuni* strain 81-176, while control animals (in total n = 11) were challenged with 0.3 ml PBS only at day 0 and 1. Animals of both groups were matched by sex and age, respectively. Mice were strictly kept in a sterile environment to avoid contaminations. At 6 days post infection, mice were sacrificed by cervical dislocation and intestinal samples were taken under aseptic conditions following the method previously described [44]. Intestinal tissue samples were obtained in parallel from each mouse for further immunofluorescence assays. Tissue sample lysis, RNA extraction, cDNA synthesis as well as protein isolation are described below.

### In vitro infection assay

*Campylobacter jejuni* infection using the cell line HT-29/B6 was performed as described previously [43]. Briefly, HT-29/B6 cells were infected with a suspension of approx. 1 × 10^9^ CFU of *C. jejuni* NCTC 11168 (MOI 500). Cell monolayers were infected for 1, 5 and 24 h post infection, respectively. Gentamicin (300 ng/ml, Biochrom) protection was performed after 3 h to kill extracellular bacteria. Before harvesting, infected cells were washed with PBS 3 times and lysed directly in each well by addition of cell lysis buffer (Roboklon, Berlin, Germany) before total RNA was extracted as described below. Non-infected cells (negative control) were treated under the same conditions and harvested at same time points, respectively.

### RNA extraction, cDNA synthesis and real-time qPCR analysis

Total RNA was isolated, and quality controlled as described earlier [3, 43]. Briefly, total RNA from HT-29/B6 cells was extracted by using the Universal RNA/miRNA Purification Kit (Roboklon, Berlin, Germany) according to the manufacturer’s protocol. Total RNA from murine colonic tissue and CMT 93 cells was isolated with the miRVana Isolation Kit (Life Technologies, Germany). The cDNA was synthesised from individual cell or tissue samples as described earlier [47]. Basically, the isolated total RNA was treated with DNase I (RNase-Free) (NEB GmbH, Frankfurt a/M, Germany) to exclude residual genomic DNA. 1 µg total RNA was reverse transcribed using 200 U M-MuLV Reverse Transcriptase (Thermo Fisher Scientific), 0.2 µg random hexamers (Thermo Fisher Scientific), 200 µM of each dNTP and 1× supplied RT buffer. Control samples were treated in the same way but without M-MuLV Reverse Transcriptase to monitor the presence of genomic DNA. Quantification of mRNA as well as miRNA expression via RT-qPCR was performed as described previously [15]. Expression data was normalised with calculated geometric means of stably expressed reference genes determined beforehand using geNorm [48]. For normalisation of mRNA expression in HT-29/B6, ACTB and B2M were used as reference genes and for normalisation of miRNA expression the small RNAs SNORD44 along with SNORD47 were used as reference [49]. For normalisation of mRNA expression in mouse colon tissue HPRT and SDHA were used as reference and miRNA expression was normalised to the miRNAs miR-16 and miR-145-5p. HPRT, SDHA and B2M were used for normalisation of mRNA expression in CMT 93 cells and miRNA expression was normalised considering miR-16 and miR-145-5p as references. The relative gene expression was calculated by the ∆∆Ct method [50] as described earlier [15]. For the cDNA synthesis and RT-qPCR analysis (miR-Q) of the miR-320-family, special RT-6 primer and reverse PCR primer (Additional file 3) with corresponding base ambiguities were generated to cover the entire members of the miR-320 family shown in Fig. 1 as described earlier [51]. Oligonucleotides used in this study were all synthesised by Sigma-Aldrich (Darmstadt, Germany) and are listed in Additional file 3.

### Western blotting

Protein from colonic tissue samples of *C. jejuni* 81–176 infected secondary abiotic IL-10^−/−^ mice was isolated and quantified as previously described with a few modifications [47, 52]. Briefly, tissue was lysed in cold RIPA buffer supplemented with protease inhibitor cocktail (Thermo Fisher Scientific). Protein samples were quantified using a BCA assay (Thermo Fisher Scientific). Equal amounts of protein (30 µg) were separated by 12% sodium dodecyl sulphate-polyacrylamide gel electrophoresis and transferred onto a polyvinylidene fluoride membrane (PVDF) (GE Healthcare, Buckinghamshire, UK) via semidry blotting. After blocking in 5% (w/v) bovine serum albumin (BSA, Sigma-Aldrich) in TBST (Tris-HCl-buffer with 0.1% (v/v) tween-20) for 2 h at room temperature, the membranes were probed with the primary antibodies (Rabbit anti-ST3GAL2, Novus Biologicals, Colorado, USA), 1:500 in 3% BSA in TBST at 4 °C overnight. Membranes were then washed three times in TBST for 15 min and subsequently incubated with horseradish peroxidase (HRP)-linked anti-rabbit IgG antibody (1:5000 in 3% BSA in TBST; Cell Signalling Technology) for 2 h at room temperature. Immuno-reactive proteins were developed by using the Amersham™ ECL Select™ Western Blotting Detection Reagent (GE Healthcare). After detection of ST3GAL2, membrane was stripped and again processed for GAPDH detection with Rabbit anti-GAPDH (#5174; Cell Signalling Technology, Danvers, MA, USA, 1:2000 in 3% BSA in TBST) and HRP-linked anti-rabbit IgG antibody (1:5000 in 3% BSA in TBST; Cell Signalling Technology). Protein quantification was performed by densitometry using the software BIO-1D (Vilber Lourmat, Marne-la-Vallée, France). Experiments were repeated at least five times.

### Immunofluorescence

For immunofluorescence detection, mouse colon sampling and immunostaining were performed as described earlier with few modification [25, 44, 53]. Briefly, mouse colon samples were immediately fixed in 5% formalin and embedded in paraffin before serial sections were cut. 5 µm of paraffin sections were deparaffinated in Roticlear (Carl Roth, Karlsruhe, Germany) and rehydrated through a graded series of ethanol followed by rinsing in distilled water and PBS (pH 7.4, Sigma-Aldrich). Non-specific binding was blocked with 1% (v/v) BSA (Sigma-Aldrich) in PBST (0.1% (v/v) Tween-20 in PBS) for 1 h at room temperature. Thereafter, sections were incubated with a 1:50 dilution of primary antibody, Rabbit anti-ST3GAL2 (Novus Biologicals), in PBST containing 1% (v/v) BSA overnight at 4 °C. Negative controls were performed without using the primary antibody. After three washes with PBS for 5 min, the primary antibody was detected with goat anti-rabbit IgG DyLight 594 (1: 400, #35561; Thermo Fisher Scientific) for 1 h at room temperature followed by two washing steps with PBS. Nuclei were counterstained with 200 ng/ml 4′, 6-diamidin-2-phenylindol (DAPI, Sigma-Aldrich) in PBS by 3 min incubation at room temperature. Subsequently, slides were washed in PBS and mounted with Prolong™ Diamond Antifade Mountant (Life Technology). Fluorescence microscopy was performed using a Leica DMI6000B inverted microscope and the Leica LAS-X software (Leica, Wetzlar, Germany). Immunofluorescence images were taken under identical microscope and camera settings. Images were taken with background-subtraction and at least four random areas per section and more than ten images per area were selected. Experiments were carried out with three biological replicates.

### RNAi and luciferase reporter assay

CMT 93 cells were cultured as described above and transfected via electroporation using the Nucleofector Technology (Lonza AG, Köln, Germany) as previously described with some modifications [3]. For nucleofection, 1 × 10^6^ cells were used together with 50 pmol of hsa-miR-615 miRVana miRNA mimics (#4464066; Fisher Scientific, Schwerte, Germany) or 50 pmol non-targeting siRNA (D-001810-03-05, Dharmacon Lafayette, CO, USA) as control. Twenty-four hours after transfection, cells were washed with PBS and lysed for RNA isolation.

Dual luciferase reporter assays were performed according to the previous study [15]. For generation of reporter plasmids, the identified target site of murine ST3GAL2 was amplified using the hybridised oligonucleotides NotI-mmuST3GAL2-ts-sense and XbaI-mmuST3GAL2-ts-antisense obtained from Sigma-Aldrich (Additional file 3). The target site was cloned in pTK-Gluc (NEB GmbH) using the restriction enzymes NotI and XbaI (NEB GmbH). The endotoxin-free reporter plasmid (pTKGmST3GAL2) was produced for transfection using NucleoBond Xtra Midi Plus EF (Macherey-Nagel GmbH & Co. KG, Düren, Germany). CMT 93 cells were transfected using 1.3 µg of pTKGmST3GAL2 and 200 ng normalisation plasmid (pTK-Cluc, NEB GmbH) along with 150 pmol Pre-miR miRNA Precursor hsa-miR-615 (Life Technologies) or 150 pmol non-sense miRNA Pre-miR miRNA Precursor Negative Control #1 (Life Technologies) as negative control. Gaussia/Cypridina Luciferase activity was detected three times for at least four independent experiments by using the Biolux Assay Kits (NEB GmbH).

### In silico-analysis

For predicting miRNAs that mutually target MUC2 and TFF3, miRmap analysis [23] was performed using a score of ≥ 50 and lists of targets were intersected using Cytoscape [54] as described earlier [24, 52]. To emphasise the biological relevance, only miRNAs were further considered that were evolutionary conserved among human, mouse and rat (Additional file 1). After evaluating anticorrelated expression between predicted miRNAs and MUC2/TFF3 in HT-29/B6, miR-125a-5p and miR-615-3p were selected for further analysis. Their targets were predicted by performing another round of miRmap analysis and lists of targets were intersected by Cytoscape (Additional file 1). KEGG pathway enrichment was performed using Cytoscape [54] and ClueGO [55]. Functionally grouped gene ontologies and pathways were determined as described previously [52] using following settings: significant KEGG pathways enrichment (*P* < 0.05), correction method: Bonferroni step down, number of genes ≥ 4, min percentage ≥ 10.0 and *Kappa* Score ≥ 0.3.

### Statistical analysis

The unpaired t-test was used in this study to compare each treatment with control. All tests were conducted applying GraphPad Prism version 6.00 (GraphPad Software, La Jolla California USA, www.graphpad.com). Asterisks in figures summarise P values (*P ≤ 0.05; **P ≤ 0.01; ***P ≤ 0.001; ****P ≤ 0.0001).

## Supplementary Information

**Additional file 1.** Raw data of in silico analysis. Sheet 1 shows the lists of miRNAs that target MUC2 and TFF3 as well as the intersection. Sheet 2 and 3 show the miRmap analysis of the mmu-miR-615-3p and mmu-miR-125a-5p targets. Sheet 4 shows the intersection of targets.

**Additional file 2.** Gene enrichment analysis by means of ClueGO analysis.

**Additional file 3.** Oligonucleotides used in this study. Sheet 1 shows the oligonucleotide sequences of mRNAs and sheet 2 shows the oligonucleotide sequences of the selected region of ST3GAL2. Sheet 3 shows the oligonucleotide sequences of miRNAs.

**Additional file 4.** Immunofluorescence detection of ST3GAL2 in colonic tissue of secondary abiotic IL-10^−/−^ mice upon *C. jejuni* 81–176 infection. (a, b) ST3GAL2 immunofluorescence in colonic tissue of non-infected mice out of two individual experiments (left panels: scale bar 100 μm, right panels: scale bar 25 μm). (c, d) Immunostaining of ST3GAL2 performed twice individually in colonic tissue of *C. jejuni* 81–176 infected mice (left panels: scale bar 100 μm, right panels: scale bar 25 μm). (e) Negative controls in serial sections on the same tissue area shown in Fig. 4A (left), supplementary Fig. a (middle) and Fig. b (right) (scale bar 25 μm). (f) Negative controls in serial sections for the same tissue area shown in Fig. 4B and supplementary Fig. c & d, respectively, (scale bar 25 μm). ST3GAL2 was immunofluorescently labelled in red and nuclei was stained with DAPI in blue.

## Data Availability

All data generated or analysed during this study are included in this published article.
