# Supplementary figures and images for: The glycosyltransferase ST3GAL2 is regulated by miR-615-3p in the intestinal tract of Campylobacter jejuni infected mice
Source: Gut Pathog. 2021 Jun 28;13:42. doi: 10.1186/s13099-021-00437-1 (PMC8240225; doi:10.1186/s13099-021-00437-1)

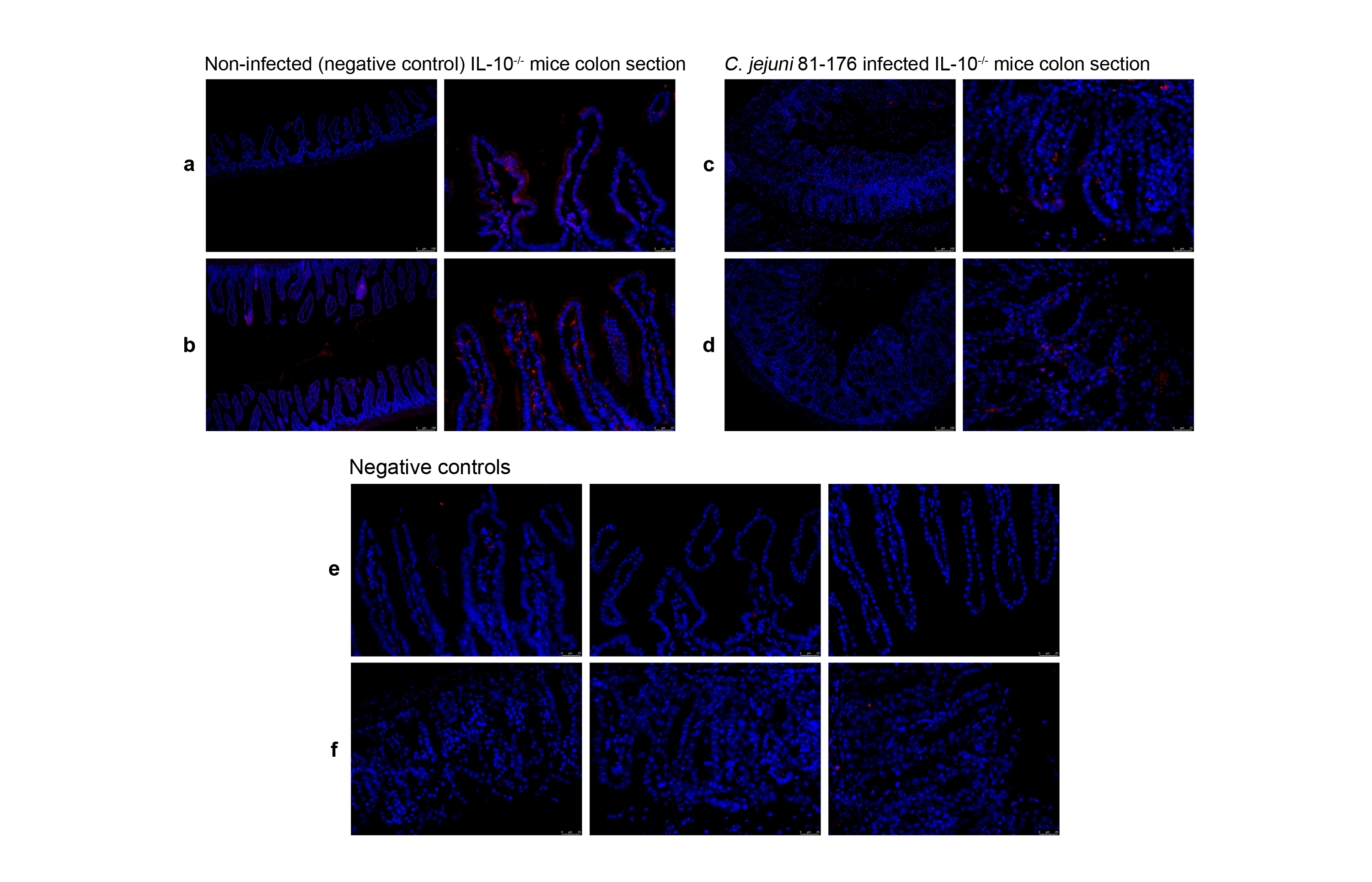

Supplement: Supplementary file 4 — Additional file 4. Immunofluorescence detection of ST3GAL2 in colonic tissue of secondary abiotic IL-10−/− mice upon C. jejuni 81–176 infection. (a, b) ST3GAL2 immunofluorescence in colonic tissue of non-infected mice out of two individual experiments (left panels: scale bar 100 μm, right panels: scale bar 25 μm). (c, d) Immunostaining of ST3GAL2 performed twice individually in colonic tissue of C. jejuni 81–176 infected mice (left panels: scale bar 100 μm, right panels: scale bar 25 μm). (e) Negative controls in serial sections on the same tissue area shown in Fig. 4A (left), supplementary Fig. a (middle) and Fig. b (right) (scale bar 25 μm). (f) Negative controls in serial sections for the same tissue area shown in Fig. 4B and supplementary Fig. c & d, respectively, (scale bar 25 μm). ST3GAL2 was immunofluorescently labelled in red and nuclei was stained with DAPI in blue. [file 13099_2021_437_MOESM4_ESM.tif]
